# Ferroptosis-related gene MAPK3 is associated with the neurological outcome after cardiac arrest

**DOI:** 10.1371/journal.pone.0301647

**Published:** 2024-06-17

**Authors:** Hong xiang Hou, Li Pang, Liang Zhao, Jihong Xing

**Affiliations:** 1 Department of Emergency, The First Hospital of Jilin University, Changchun, China; 2 Rehabilitation Department, The First Hospital of Jilin University, Changchun, China; Columbia University Irving Medical Center, UNITED STATES

## Abstract

**Background:**

Neuronal ferroptosis is closely related to the disease of the nervous system, and the objective of the present study was to recognize and verify the potential ferroptosis-related genes to forecast the neurological outcome after cardiac arrest.

**Methods:**

Cardiac Arrest-related microarray datasets GSE29540 and GSE92696 were downloaded from GEO and batch normalization of the expression data was performed using “sva” of the R package. GSE29540 was analyzed to identify DEGs. Venn diagram was applied to recognize ferroptosis-related DEGs from the DEGs. Subsequently, The Gene Ontology (GO) and Kyoto Encyclopedia of Genes and Genomes (KEGG) enrichment analysis were performed, and PPI network was applied to screen hub genes. Receiver operating characteristic (ROC) curves were adopted to determine the predictive value of the biomarkers, and the GSE92696 dataset was applied to further evaluate the diagnostic efficacy of the biomarkers. We explore transcription factors and miRNAs associated with hub genes. The “CIBERSORT” package of R was utilized to analyse the proportion infiltrating immune cells. Finally, validated by a series of experiments at the cellular level.

**Results:**

112 overlapping ferroptosis-related DEGs were further obtained via intersecting these DEGs and ferroptosis-related genes. The GO and KEGG analysis demonstrate that ferroptosis-related DEGs are mainly involved in response to oxidative stress, ferroptosis, apoptosis, IL-17 signalling pathway, autophagy, toll-like receptor signalling pathway. The top 10 hub genes were selected, including HIF1A, MAPK3, PPARA, IL1B, PTGS2, RELA, TLR4, KEAP1, SREBF1, SIRT6. Only MAPK3 was upregulated in both GSE29540 and GAE92696. The AUC values of the MAPK3 are 0.654 and 0.850 in GSE29540 and GSE92696 respectively. The result of miRNAs associated with hub genes indicates that hsa-miR-214-3p and hsa-miR-483-5p can regulate the expression of MAPK3. MAPK3 was positively correlated with naive B cells, macrophages M0, activated dendritic cells and negatively correlated with activated CD4 memory T cells, CD8 T cells, and memory B cells. Compared to the OGD4/R24 group, the OGD4/R12 group had higher MAPK3 expression at both mRNA and protein levels and more severe ferroptosis.

**Conclusion:**

In summary, the MAPK3 ferroptosis-related gene could be used as a biomarker to predict the neurological outcome after cardiac arrest. Potential biological pathways provide novel insights into the pathogenesis of cardiac arrest.

## 1. Introduction

Cardiac Arrest (CA) is a severe clinical emergency with high mortality and disability. In 2010 the global average annual incidence of out-of-hospital cardiac arrest (OHCA) in adults was reported to be 55 per 100,000 residents [1]. Later research data shows that the incidence of OHCA in all ages is 88.8 per 1000,00 population [2, 3] and 49 per 100,000 inhabitants in Europe [4], approximately 290,000 adults experience in-hospital cardiac arrest (IHCA) annually in the United States [5]. However, the incidence rate of OHCA in China is 71.2 per 100,000 people [6, 7]. Cardiopulmonary resuscitation (CPR) is an emergency treatment after CA, and the earlier it is performed, the better the prognosis. Brain injury, systemic ischemia-reperfusion, myocardial dysfunction, and persistent precipitating pathophysiology after Restoration of Spontaneous Circulation (ROSC) are known as post-cardiac arrest syndrome [8]. Post-resuscitation brain injury, a common sequela after cardiac arrest, is a primary cause of death and long-term disability. Accurate prediction is a crucial aspect of the management of comatose patients after CA, and recent research suggests that in addition to neuron-specific enolase (NSE) and S100B, glial fibrillary acidic protein (GFAP), neurofilament light (NfL), ubiquitin carboxy-terminal hydrolase L1 (UHC-L1), and tau protein (tau) and other indicators (glasgow coma scale, pupil size and its light reflex, CT and MRI imaging, electroencephalography (EEG), and so on) can also be used to predict neurological outcome after CA [9]. NSE, ease of sampling and interpretation, low effect of sedation and quantitative measurement of brain damage are advantages over other methods [10]. The major limitations are the release of biomarkers from extracerebral sources, as NSE is also present in erythrocytes [10–12]. The specificity of S100B was lower than that of NSE [13]. Compared with other predictors after cardiac arrest, biomarkers(NSE, S100B, GFAP, NfL, UHC-L1, tau and so on) are not affected by sedation or paralysis, but have limitations including potential interference from extracerebral sources, the use of different measurement techniques, and reduced availability compared with other index tests [12]. Like pupillary reflex, corneal reflex is susceptible to interference from sedatives, opioids or neuromuscular blocking agents [12]. EEG may be affected by sedation, metabolic derangements, and body temperature. The main limitation is that most clinicians will judge the predictor subjectively, as quantification of avGWR (CT) and ADC (DWI) requires specialised expertise and software that is inaccessible to most clinicians at the bedside [11]. Guidelines and experts recommend an approach that combines a number of different methods. It is well worthwhile to explore additional biomarkers for assessing neurological prognosis after cardiac arrest. Physicians and researchers continue to work on ways to improve neurological outcomes and quality of life for patients who have suffered cardiac arrest. Ferroptosis is a new type of procedural death that has been discovered and studied in recent years, with increasing evidence that it is associated with a wide range of conditions, including brain damage after cardiac arrest. So we tried to use bioinformatics to explore the relationship between ferroptosis-related genes and cerebral ischemia-reperfusion injury after cardiac arrest.

A new form of cell death distinct from apoptosis, miscellaneous necrosis and autophagy in morphology, biochemistry, and genetics; it was first defined as ferroptosis in 2012 by Stockwell et al [14]. Since then, there has been increasing interest in the process and mechanism of ferroptosis, and more and more research has found that neuronal ferroptosis is closely related to nervous system diseases, such as Alzheimer’s Disease (AD), Parkinson’s Disease (PD), Huntington’s Disease (HD), traumatic brain injury, stroke, intracerebral hemorrhage, ischemia-reperfusion injury [15]. Zifeng Huang et al. determined that unusual overexpression of SNX5 promotes ferroptosis in PD [16]. A finding testified that iron is closely related to mitochondrial disorders and injury in mouse models of human HD [17]. The occurrence of iron deposition is strongly associated with memory loss and cognitive decline in people with Alzheimer’s disease [18]. A recent study suggests that ACSL4 aggravates ischemic stroke via accelerating ferroptosis-induced brain injury and neuroinflammation [19]. Chiwei Peng et al. demonstrated that dauricine, via upregulating glutathione peroxidase 4 (GPX4) and glutathione reductase (GSR) co-expression, could suppress neuronal ferroptosis and relieve brain injury after intracerebral hemorrhage [20]. As an iron uptake inhibitor, Ferristatin II plays a neuroprotective role in traumatic brain injury by alleviating ferroptosis [21]. Qing-zhang Tuo et al. reported that thrombin induces ferroptosis during cerebral ischemia reperfusion [22]. Chen C et al. demonstrated that by inhibiting of the ACSL4/GPx4 pathway mediated ferroptosis, Alda-1 may have the potential to reduce brain injury after CPR in swine [23]. Some studies have found that sodium octanoate significantly alleviated heart and brain injury after TCA, which may be related to inhibition of cell apoptosis and GPX4-mediated ferroptosis [24]. A series of experiments using a rat model of cardiac arrest showed that UAMC-3203 and DFO improved cardiac function after ROSC, which could be a novel potential target for the treatment of PRMD [25]. Hongbo Wu et al. using a porcine CA/CPR model found that Alda-1 treatment was effective in alleviating lung injury after CA/CPR through the inhibition of cell apoptosis and ferroptosis [26]. However, the specific mechanism of ferroptosis in cerebral ischemia reperfusion after cardiac arrest remains unclear.

To our knowledge, there are currently no bioinformatics-based studies of the mechanism of ferroptosis genes in the area of neurological outcomes after CA. Therefore, data mining and data analysis techniques were applied to screen for differentially expressed genes (DEGs) in both CPC (1–2) and CPC (3–5) patients. The Cerebral Performance Category (CPC) scale was used to assess neurological outcome: patients with a CPC score of 1 had no or mild neurological disability, CPC score of 2 had moderate disability, CPC score of 3 had severe disability, CPC score of 4 were in a coma or vegetative state, CPC score of 5 were death. Those with CPC 1–2 were defined as having a favourable prognosis of neurological function, and those with CPC3-5 were defined as having an unfavourable prognosis of neurological function [27, 28]. Then, the identified DEGs were cross-referenced with the ferroptosis database to acquire ferroptosis-related DEGs. Hence, the objective of the current study was to identify potential biomarkers associated with ferroptosis and neurological outcomes after CA, in order to provide novel insights into the diagnosis, treatment and prognosis of CA.

## 2. Materials and methods

### 2.1 Data normalization and differential expression analysis

The “sva” of R package was used to proceed batch normalization of the expression data, then the normalized data were separated separately for differential expression analysis. The R package “limma” was used to screen for DEGs with a threshold of FC (fold changes) > 1 and p-value < 0.05. To visualise these DEGs, volcano plots and heatmaps were generated using the R packages “ggplot2” and “pheatmap” respectively [29].

### 2.2 Identification of differential expression genes associated with ferroptosis

Recently, the ferroptosis database was upgraded from V1 to V2 [30]. A total of 431 ferroptosis-related genes were downloaded from the FerrDb V2 database, including drivers, suppressors and 9 markers according to previous studies [31, 32] (S1 Data). The Venn diagram web tool from Sangerbox (**http://vip.sangerbox.com/**) was applied to identify ferroptosis-related DEGs from the DEGs in GSE29540.

### 2.3 Functional and pathway enrichment analysis

The Gene Ontology (GO) and Kyoto Encyclopedia of Genes and Genomes (KEGG) enrichment analyses of the DEGs were performed using the ‘clusterProfiler’[33], ‘org.Hs.eg.db’, and ‘enrichplot’ packages of the R statistical software(p<0.05). GO analysis included three functional categories, biological processes (BP), cell component (CC), and molecular function (MF) [34]. KEGG analysis was applied to probe the possible pathways [35].

### 2.4 Protein–Protein interaction network analysis of ferroptosis-related DEGs

A extremely popular online database STRING (https://string-db.org/) was applied to search for the proteins interactions with interaction scores > 0.4 [36]. Cytoscape software was utilized to visualize the PPI network [37]. A novel Cytoscape plugin, cytoHubba, was applied to screen the hub genes of the PPI network, and ultimately the ten genes were selected as hub genes using the maximal clique centrality (MCC) algorithm.

### 2.5 Value of key ferroptosis-related DEGs as biomarkers to predict the neurological outcome after cardiac arrest

Receiver operating characteristic (ROC) curve was adopted to determine the predictive value of the biomarkers. The area under the ROC curve (AUC) was calculated to determine the diagnostic performance of the hub biomarker on the GSE29540 dataset. Afterward, the GSE92696 dataset was applied to further evaluate the diagnostic efficacy of the biomarkers.

### 2.6 Transcription factor-hub gene network and miRNAs associated with hub genes

The visual online platform NetworkAnalyst 3.0 was applied to predict the TF of the hub genes. We apply miRDB, starBase v2.0, miRTargets database to screen the target genes of the miRNAs.

### 2.7 Immune infiltration analysis

The “CIBERSORT” package in R was utilized to analyse the proportion of infiltrating immune cells in both CPC (1–2) and CPC (3–5) [38]. Simultaneously, We use the “corrplot” package of R to proceed the correlation analysis of the immune correlation between the gene biomarkers and the immune cells. The result was visualized as a violin plot using the “vioplot” package.

### 2.8 Correlation analysis of ferroptosis-related DEGs and infiltrating immune cells

Pearson correlation analysis was utilized to examine the correlation between biomarkers and immune cell infiltration. The results of the correlation analysis were presented in the violin plot using the “ggplot2” software package of R.

### 2.9 Cell culture and oxygen-glucose deprivation and reperfusion

Human neuroblastoma cells, SH-SY5Y, derived from human bone marrow, can differentiate into mature neuronal phenotypes and express neuron-specific markers. SH-SY5Y has been widely used in the in vitro study of Parkinson’s disease, Alzheimer’s disease, cerebral ischemia-reperfusion injury and other diseases [39–41]. So we chose SH-SY5Y to construct an in vitro OGD/R model. SH-SY5Y neuroblastoma cells from the Jilin Provincial Key Laboratory of Cerebrovascular Disease were cultured in high-glucose DMEM medium (Procell, Wu Han, China) supplemented with 10% fetal bovine serum (FBS) (Procell, Wu Han, China), 1% penicillin-streptomycin and 1% glutamine under a humidified atmosphere of 5% CO2 and 95% air at 37°C. When the cell density reached 70–80%, the culture medium was changed glucose-free DMEM (Gibco, Gaithersburg, USA) medium without FBS. The cells then were transferred to an anaerobic culture bag (MGC: Mitsubishi Gas Chemical) [42], which contained an anaerobic gas producing bag (MGC) and an anaerobic indicator (MGC) at 37°C. The Anaerobic Indicator changes from blue to pink when the oxygen level falls below 1%, indicating anoxic conditions. After 4 h, the anaerobic equipment was removed and the culture medium was changed back to complete DMEM under a humidified atmosphere of 5% CO2 and 95% air at 37°C for 12 h and 24 h [41].

### 2.10 Cell viability assays

The cell counting kit-8 (CCK8, invigentech, USA) assay was used to determine cell viability according to the manufacturer’s instructions. Briefly, SY5Y cells in the logarithmic growth phase were cultured onto a 96-well plate at a density of 3×10^3^ cells/well in 100 ul culture medium and grown overnight. Then, Cells were subjected to Oxygen-Glucose Deprivation for 4 hours and reperfusion for 12,24 hours. Next, 10 ul of CCK8 solution was added to each well and continued to incubate for another 1h at 37°C. The absorbance (OD) value of each well at 450 nm was determined using a multifunctional automatic enzyme labeller (BioTek, USA).

### 2.11 Reactive Oxygen Species (ROS) assay and mitochondrial membrane potential assay (JC-1)

The reactive oxygen species (ROS) assay was used to assess intracellular ROS according to the manufacturer’s instructions. Briefly, SY5Y cells in the logarithmic growth phase were cultured onto per 6-cm diam petri dish at a density of 1×10^6^ cells in a 3ml culture medium and cultured for 24 hours. Then, cells were subjected to oxygen-glucose deprivation for 4 hours and reperfusion for 12, 24 hours. Then, cells were collected to obtain cell suspension and washed with PBS. 5×10^5^ cells were incubated in 10 μM DCFH-DA solution (Beyotime, China) at 37°C for 20 min. The cells were washed three times with Serum-free medium, ROS production in cells was determined using a flow cytometer (BD, Mountain View, CA, USA). The data was analyzed using FlowJo software version 10.8.1 (TreeStar, Ashland, OR, USA). Meanwhile, SY5Y cells in the logarithmic growth phase were cultured onto a 24-well plate crawl piece at a density of 3×10^4^ cells/well in 500 ul culture medium and cultured for 24 hours. Then, Cells were subjected to Oxygen-Glucose Deprivation for 4 hours and reperfusion for 12, 24 hours. Cells were washed with PBS. Then, cells were incubated in 10 μM DCFH-DA solution (Beyotime, China) or JC-1 working fluid (Elabscience, Wuhan, China) at 37°C for 20 min. The cells were washed three times with serum-free medium or 1×JC-1 assay buffer and kept out of the light during this time. The cell crawlers were removed and placed on a microscope slide. Finally, fluorescence was measured by laser confocal microscopy (FV1000, Olympus, Japan), Image J software was used to analyse the fluorescence intensity of each group.

### 2.12 Quantitative real-time PCR

Total RNA was extracted from SY5Y cells in accordance with the manufacturer’s instructions using the TransZol Up Plus RNA Kit (TransGen, ER501-01). Following the manufacturer’s instructions, 1ug of total RNA was reverse transcribed into cDNA using TransScript® Uni All-in-One First-Strand cDNA Synthesis SuperMix for qPCR (TransGen, AU341-02). Following the manufacturer’s instructions, qRT-PCR was carried out using PerfectStart® Green qPCR SuperMix (TransGen, AQ601-02) on an ABI StepOnePlus system (Applied Biosystems). The 2-ΔΔCT method was used for calculation of relative mRNA expression. The following primers were used: MAPK3(Forward: <monospace_wrap>CTACACGCAGCTGCAGTACATC</monospace_wrap>, Reverse: <monospace_wrap>GTGCGCTGACAGTAGGTTTGA</monospace_wrap>), GPX4(Forward: <monospace_wrap>CCGTCTGAGCCGCTTATTGAA</monospace_wrap>, Reverse: <monospace_wrap>ACACGCAACCCCTGTACTTA</monospace_wrap>), FTH1(Forward: <monospace_wrap>GGCGACGGAAGTGGTTGTTA</monospace_wrap>, Reverse: <monospace_wrap>ACAGGGTTACTGGTCAGCTCT</monospace_wrap>), TFR1(Forward: <monospace_wrap>GAATACGTT CCCCGTTGTTGA</monospace_wrap>, Reverse: <monospace_wrap>ATCCCCAGTTCCTAGATGAGCAT</monospace_wrap>), β-actin(Forward: <monospace_wrap>CCCATCTATGAGGGTTACGC</monospace_wrap>, Reverse: <monospace_wrap>TTTAATGTCACGCACGATTTC</monospace_wrap>).

### 2.13 Flow cytometric analysis

SY5Y cells in the logarithmic growth phase were cultured onto per 6-cm diam petri dish at a density of 1×10^6^ cells in a 3ml culture medium and cultured for 24 hours. Then, cells were subjected to oxygen-glucose deprivation for 4 hours and reperfusion for 12, 24 hours. Cells were harvested and primary and fluorescent secondary antibodies were used to detect the expression of the corresponding proteins. The list of antibodies is as follows: ERK1/2 (MAPK3/1, proteintech, 51068-1-AP), p-ERK1/2 (p-MAPK3/1, proteintech, 28733-1-AP), GPX4 (proteintech, 11257-1-AP), FTH1(aways, CY7085), TFR1(aways, CY6618), Fluorescein (FITC)–conjugated Affinipure Goat Anti-Mouse (proteintech, SA00003-1) and CoraLite594 –conjugated Goat Anti-Rabbit (proteintech, SA00013-4). For cell surface antigen staining, formaldehyde fixation and 4% permeabilization (Solarbio) for 15 minutes, primary antibody incubation for 1 hour, followed by fluorescent secondary antibody incubation for half an hour for the corresponding intracellular proteins. For staining of intracellular antigens, the membrane was fixed and permeabilized with 4% permeabilization (Solarbio) for 15 minutes, broken down with methanol (Solarbio) for 1 hour, incubated with primary antibody for 1 hour and then incubated with fluorescent secondary antibody for half an hour to stain the corresponding intracellular proteins. All samples were collected on a flow cytometer (BD, Mountain View, CA, USA). The data was analyzed using FlowJo software version 10.8.1 (TreeStar, Ashland, OR, USA).

### 2.14 Immunofluorescence

SY5Y cells in the logarithmic growth phase were cultured onto a 24-well plate crawl piece at a density of 3×10^4^ cells/well in 500 ul culture medium and cultured for 24 hours. Then, Cells were subjected to Oxygen-Glucose Deprivation for 4 hours and reperfusion for 12, 24 hours. All groups were fixed with 4% paraformaldehyde (Solarbio) for 20 minutes, 0.5% Triton X-100 (Beyotime) for 15 minutes, 5% BSA (Beyotime) for half an hour, primary antibody ERK1/2 (MAPK3/1, proteintech, 51068-1-AP), p-ERK1/2 (p-MAPK3/1, proteintech, 28733-1-AP), GPX4 (proteintech, 11257-1-AP), FTH1(aways, CY7085)) incubated overnight, and fluorescent secondary antibody incubated for 1 hour. The slides were then sealed with DAPI-containing sealer and photographed using a laser confocal microscopy (FV1000, Olympus, Japan), Image J software was used to analyse the fluorescence intensity of each group.

### 2.15 MDA, GSH and Fe^2+^ measurement

According to the instructions of the malondialchehyche (MDA activity assay kit (Nanjing Jiancheng Bioengineering Institute), glutathione (GSH) assay kit (Nanjing Jiancheng Bioengineering Institute) and iron colorimetric assay Kit (Nanjing Jiancheng Bioengineering Institute), the levels of MDA, GSH and Fe^2+^ were detected in each group separately.

## 3. Statistical analysis

Bioinformatic analyses were executed applying R v.4.1.3. The Wilcoxon rank-sum test was applied to compare two groups. A two-tailed p-value < 0.05 was identified as significant differences. Experiments were performed in triplicate. Statistical analyses were conducted using GraphPad Prism 9.0.

## 4. Results

### 4.1 Differential expression analysis and ferroptosis-related differential expression genes

**Fig 1:**
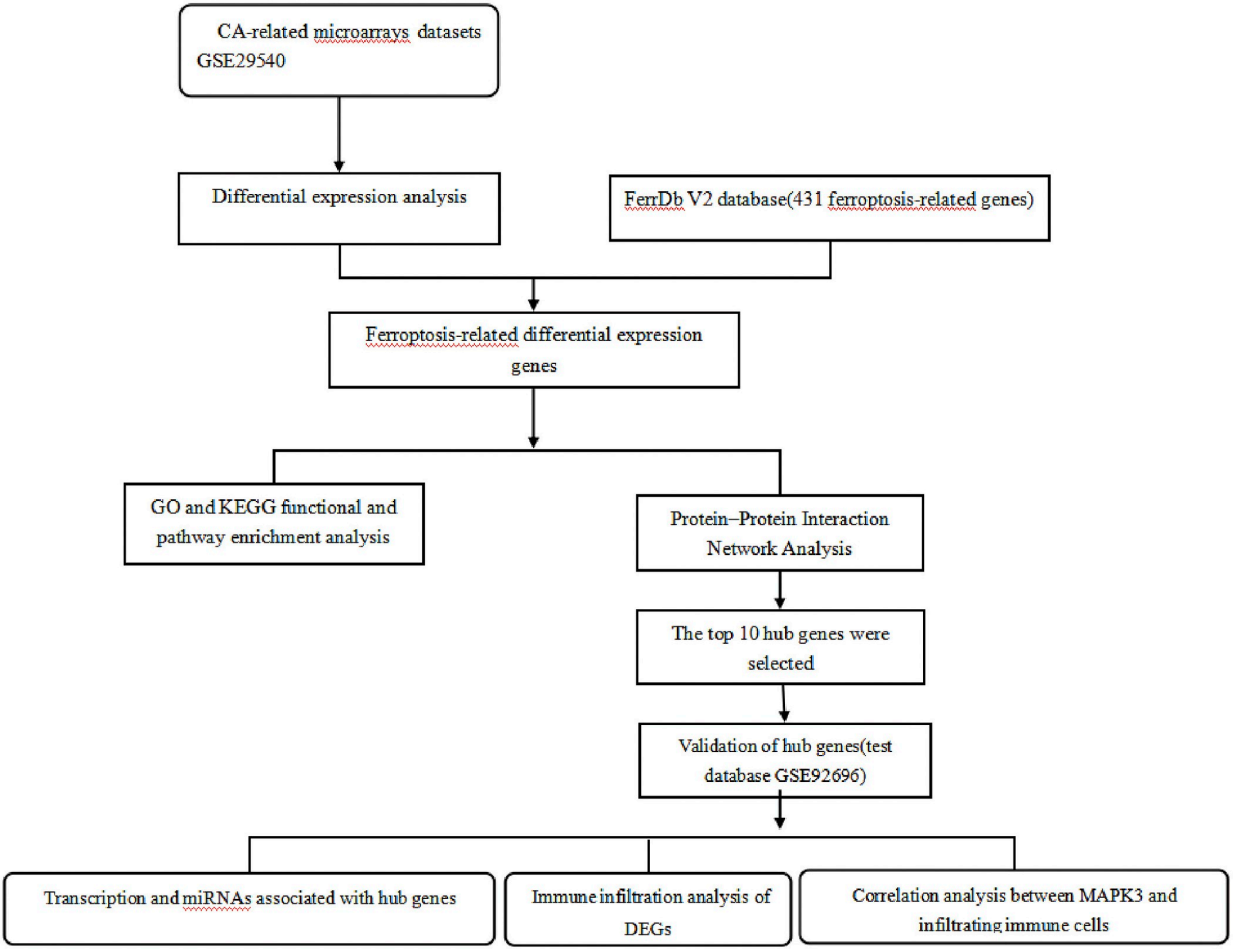
Flowchart.

The flowchart of this study is shown in Fig 1. We downloaded the CA-related microarray datasets GSE29540 and GSE92696 from GEO (Gene Expression Omnibus), including the patients’ mRNA expression profiles and clinical information (Table 1). GSE29540 and GSE92696 serve as the training datasets and verification datasets respectively. In the figure, who CPC score 1–2 display “low”, who CPC score 3–5 display “high”.CA-related microarrays datasets GSE29540Differential expression analysisFerrDb V2 database(431 ferroptosis-related genes)Ferroptosis-related differential expression genesGO and KEGG functional and pathway enrichment analysisProtein–Protein Interaction Network AnalysisThe top 10 hub genes were selectedValidation of hub genes(test database GSE92696)Transcription and miRNAs associated with hub genes Immune infiltration analysis of DEGsCorrelation analysis between MAPK3 and infiltrating immune cellsCA-related microarrays datasets GSE29540Differential expression analysisFerrDb V2 database(431 ferroptosis-related genes)Ferroptosis-related differential expression genesGO and KEGG functional and pathway enrichment analysisProtein–Protein Interaction Network AnalysisThe top 10 hub genes were selectedValidation of hub genes(test database GSE92696)Transcription and miRNAs associated with hub genes Immune infiltration analysis of DEGsCorrelation analysis between MAPK3 and infiltrating immune cells.

**Table 1:** Dataset information.

source	Dataset	Platform	Total	Low (CPC 1–2)	High(CPC 3–5)
blood	GSE29540	GPL13218	140	84	56
blood	GSE92696	GPL10558	22	10	12

**Fig 2:**
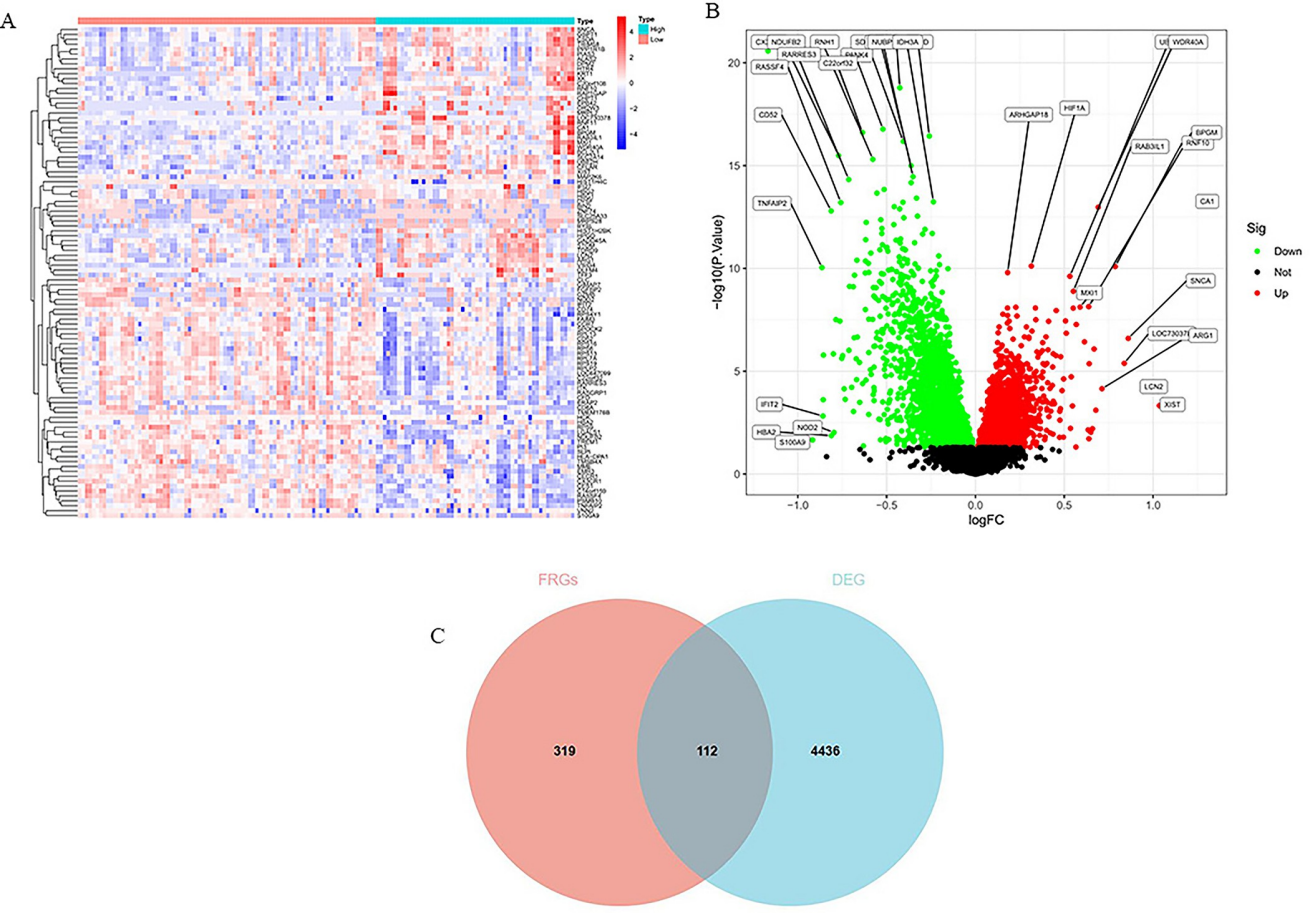
DEGs between good outcome and poor outcome of neurological function. (A) The heatmap shows top 50 upregulated and downregulated DEGs. The X-axis delegates the sample type, and the Y-axis delegates the DEGs. (B) Volcano plot exhibit DEGs,the red points show upregulated genes, and the green points represent downregulated genes. The X-axis delegates the logFC, and the Y-axis represents the -log10 (P.Value). P values < 0.05 was identified as significant differences. (C) Venn diagram show ferroptosis differentially expressed genes. Ferroptosis differentially expressed genes were selected by intersecting ferroptosis dataset with GSE29540 DEGs.

4548 DEGs were selected from GSE29540 by differential expression analysis, including 2440 downregulated and 2108 upregulated genes (S2 and S3 Data). The top fifty upregulated and downregulated DEGs were visualized via a heatmap and volcano plot (Fig 2A and 2B). 112 overlapping ferroptosis-related DEGs were further obtained via intersecting these DEGs and ferroptosis-related genes (Fig 2 C and S4 Data).

### 4.2 Functional and pathway enrichment analysis

**Fig 3:**
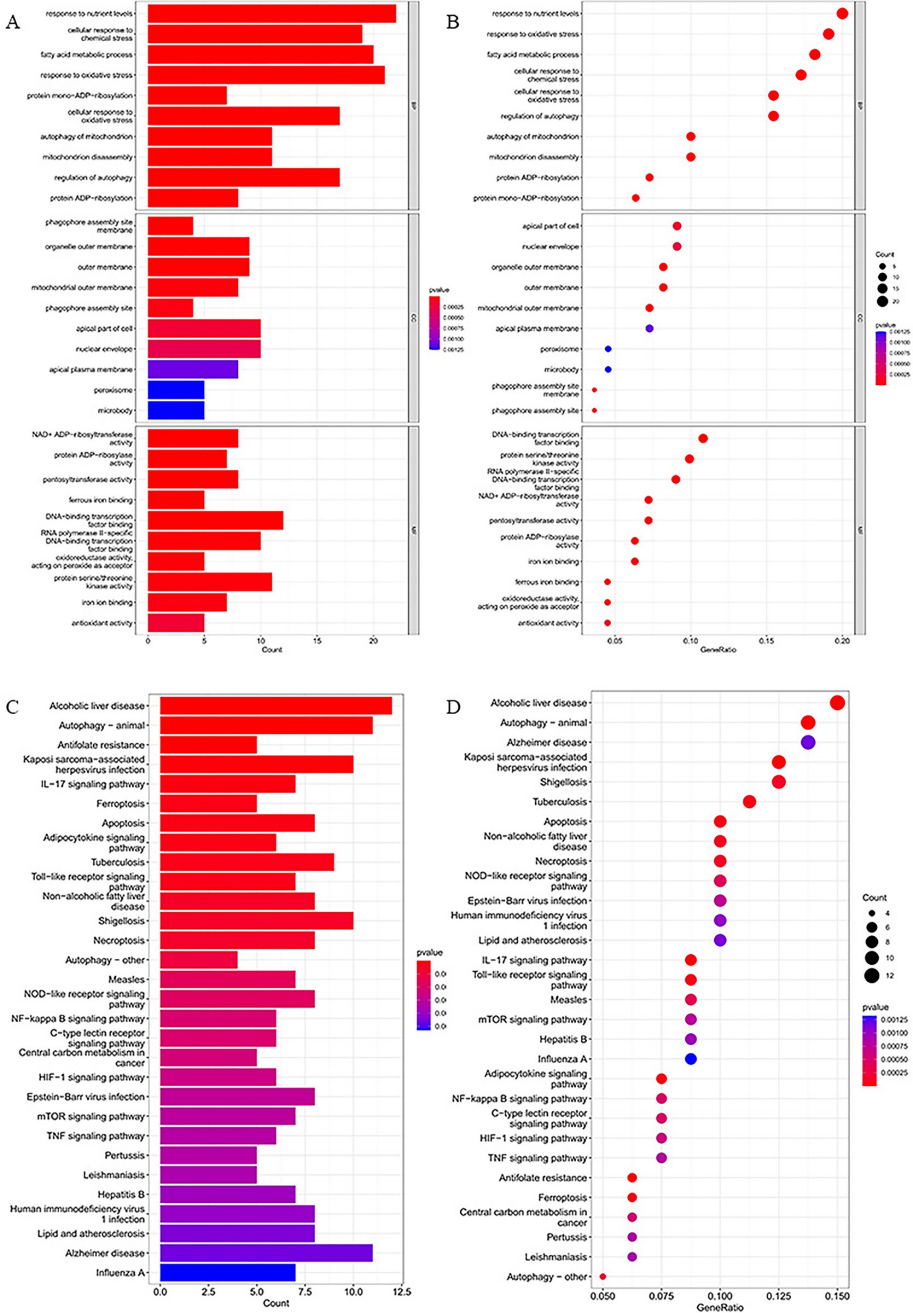
GO and KEGG enrichment analyses of 112 ferroptosis-related DEGs. The (A) bar plot and (B) bubble of Significant GO analysis. The (C) bar plot and (D) bubble show Significant KEGG pathways.

We conducted GO and KEGG enrichment analyses to investigate the potential biological processes and pathways involved in 112 ferroptosis-related DEGs. We use the bar chart and the bubble chart to visualize the top 10 results of the GO analysis for Biological Processes (BP), Cellular Component (CC) and Molecular Function (MF) that have the smallest p-value. As we can see from the Fig 3, as for BP, ferroptosis-related DEGs were mainly involved in fatty acid metabolic process, response to oxidative stress, protein mono-ADP-ribosylation, cellular response to oxidative stress, autophagy of mitochondrion, mitochondrion disassembly, regulation of autophagy; in terms of CC, ferroptosis-related DEGs were significantly enriched in phagophore assembly site membrane, organelle outer membrane, outer membrane, mitochondrial outer membrane, phagophore assembly site, apical part of cell; in the MF analysis, ferroptosis-related DEGs were remarkably enriched in NAD+ ADP-ribosyltransferase activity, protein ADP-ribosylase activity, ferrous iron binding, DNA-binding transcription factor binding, iron ion binding, antioxidant activity. Meanwhile, the results of the KEGG enrichment analysis demonstrated that these ferroptosis-related DEGs were significantly enriched in Ferroptosis, Apoptosis, IL-17 signalling pathway, Autophagy-animal, Toll-like receptor signalling pathway, Necroptosis, NF-kappa B signalling pathway, HIF-1 signalling pathway, Alzheimer’s disease.

### 4.3 Protein-Protein interaction network establishment and identification hub genes of ferroptosis-related DEGs

**Fig 4:**
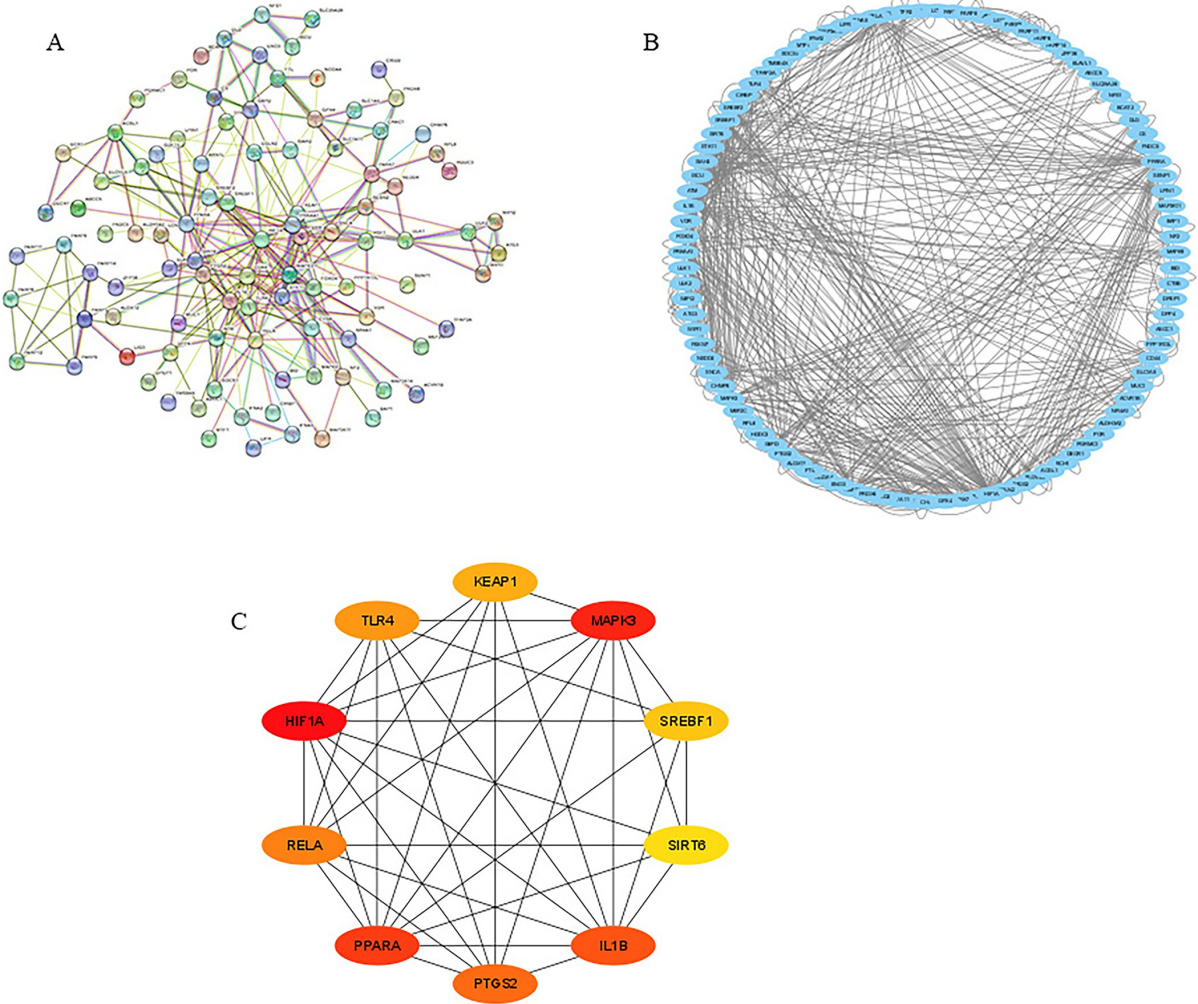
(A): PPI network of ferroptosis-related DEGs. (B):The PPI networks was visualized using Cytoscape software. (C):The top10 ferroptosis-related DEGs using MCC.

The STRING was utilized to construct a PPI network to further screen the candidate hub genes based on these ferroptosis-related DEGs. The PPI network contains 99 nodes and 302 edges (Fig 4A). Thirteen of the 112 genes were unrelated to other molecules and did not form molecular networks. Cytoscape software was used to visualize the results (Fig 4B). The top 10 hub genes were selected via the MCC algorithm of the Cytoscape CytoHubba plug-in, including HIF1A, MAPK3, PPARA, IL1B, PTGS2, RELA, TLR4, KEAP1, SREBF1, SIRT6 (Fig 4C).

### 4.4 Validation of hub genes

**Fig 5:**
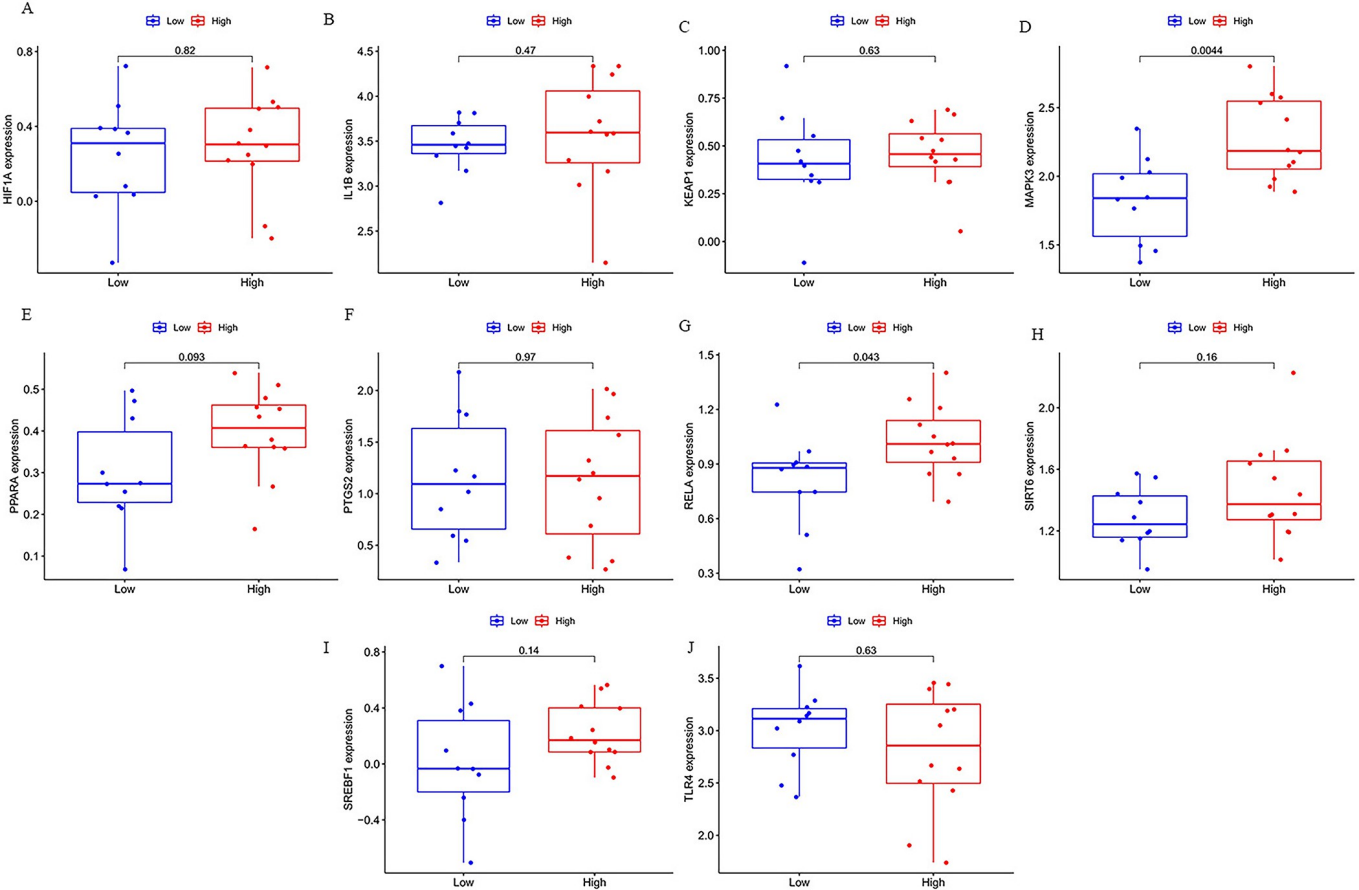
(A-J) Expression of hub geenes in GSE92696.

The GSE92696 database was utilized to verify hub gene expression. The results show that only two of the ten hub genes MAPK3, RELA had substantially different expression levels between good outcome and poor outcome in neurological function, on closer inspection, RELA is downregulated in GSE29540 and upregulated in GSE92696, only MAPK3 was upregulated in both GSE29540 and GAE92696 (p < 0.05, Fig 5 and S1 Fig in S1 File). We speculated that the ferroptosis-related gene MAPK3 might be associated with neurological prognosis after cardiac arrest.

**Fig 6:**
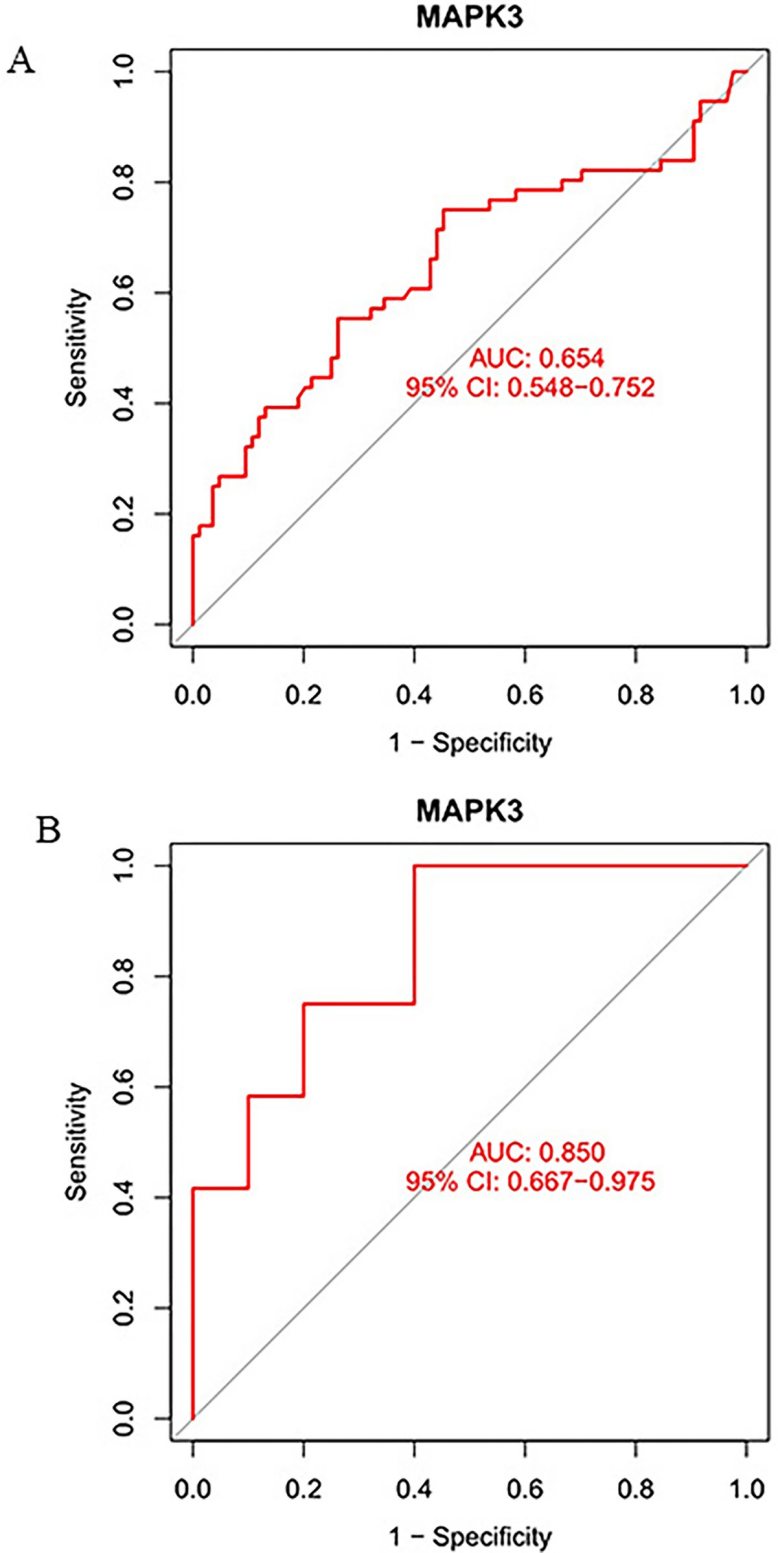
(A) ROC curve of MAPK3 in the GSE29540; (B) ROC curve of MAPK3 in another dataset of GSE92696.

The ROC analysis was applied to evaluate the potential of markers to discriminate CA patients with good neurological function outcome from CA patients with poor neurological function outcome. The results demonstrated that the AUC values of the MAPK3 in GSE29540 and GSE92696 were 0.654(95% CI:0.548–0.752) and 0.850(95% CI:0.667–0.975), respectively (Fig 6).

### 4.5 Transcription factor and miRNAs associated with hub genes

**Fig 7:**
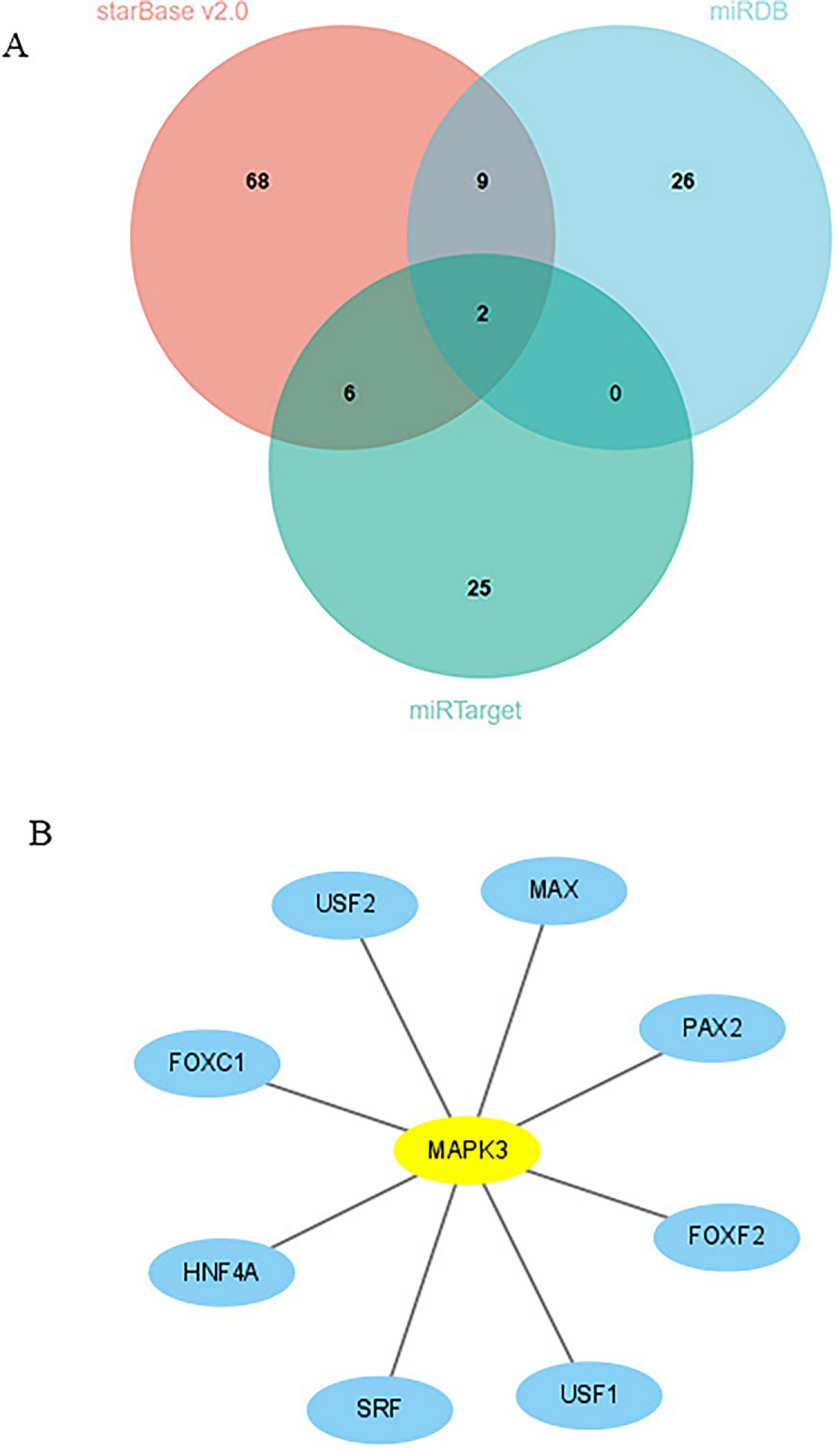
(A). Venn diagram show common miRNA. (B). Interactions MAPK3 and transcription factors.

The gene-TF network of MAPK3 is shown in Fig 7A. From the picture, USF2, MAX, PAX2, FQXF2, USF1, SRF, HNF 4A, FOXC1 interact with MAPK3. Hsa-miR-214-3p and hsa-miR-483-5p were further obtained via intersecting miRDB, starBase v2.0 and miRTargets three state database (Fig 7B and S5–S8 Data).

### 4.6 Immune infiltration analysis

**Fig 8:**
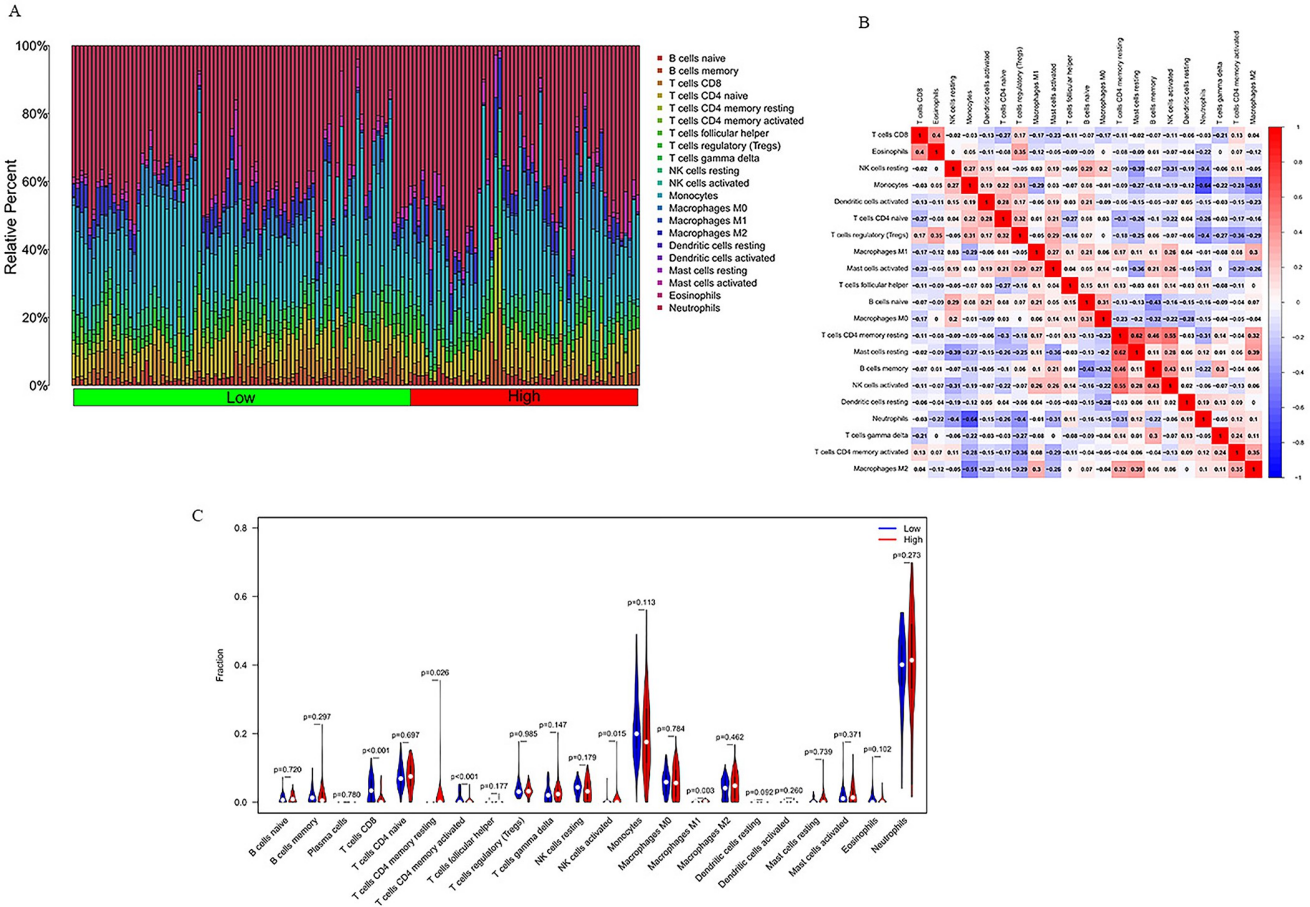
(A). Proportions of 22 types of immune cells. (B). Heatmap visualize the correlation of the infiltration of immune cells. (C). Violin plot of the proportion of 22 types of immune cell infiltrates.

The CIBERSORT algorithm was devoted to calculate the immune infiltration in CA patients. The enrichment fractions of 22 types of immune infiltrating cells in the low and high CPC scores are shown in Fig 8A. Correlation analysis demonstrated that resting mast cells and resting CD4 memory T cells had the most significant positive relationship with r = 0.62, while neutrophils and monocytes had the most significant negative correlation with r = -0.64 (Fig 8B). According to the results, 5 immune cells in the low CPC group were significantly different from those in the high CPC group, including CD8 T cells, resting CD4 memory T cells, activated CD4 memory T cells, activated NK cells, M1 Macrophages (P<0.05) (Fig 8C).

### 4.7 Correlation analysis between MAPK3 and infiltrating immune Cells

**Fig 9:**
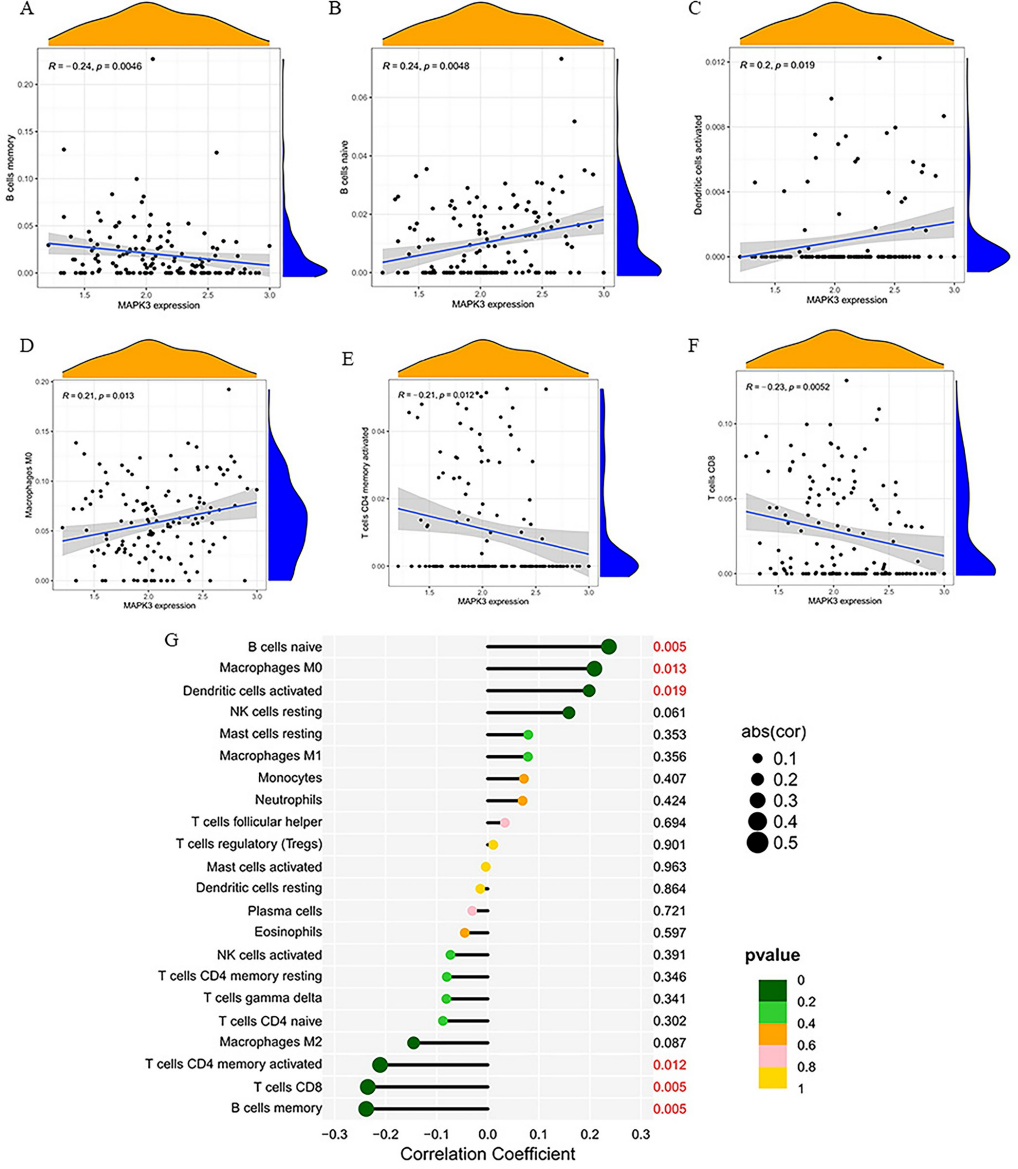
(A-G) Correlations between between MAPK3 and infiltrating immune cells.

In correlation analysis, we demonstrated that MAPK3 was positively correlated with naive B cells (r  =  0.24, P  =  0.0048), M0 macrophages (r  =  0.21, P  =  0.013), activated dendritic cells (r  =  0.2, P  =  0.019) and negatively correlated with activated CD4 memory T cells (r  = -0.21, P  =  0.012), CD8 T cells (r  =   -0.23, P  =  0.0052), memory B cells (r  =   -0.24, P  =  0.0046) (Fig 9).

### 4.8 More severe ferroptosis in OGD4/12 group than in OGD4/R24 group in SY5Y cells

**Fig 10:**
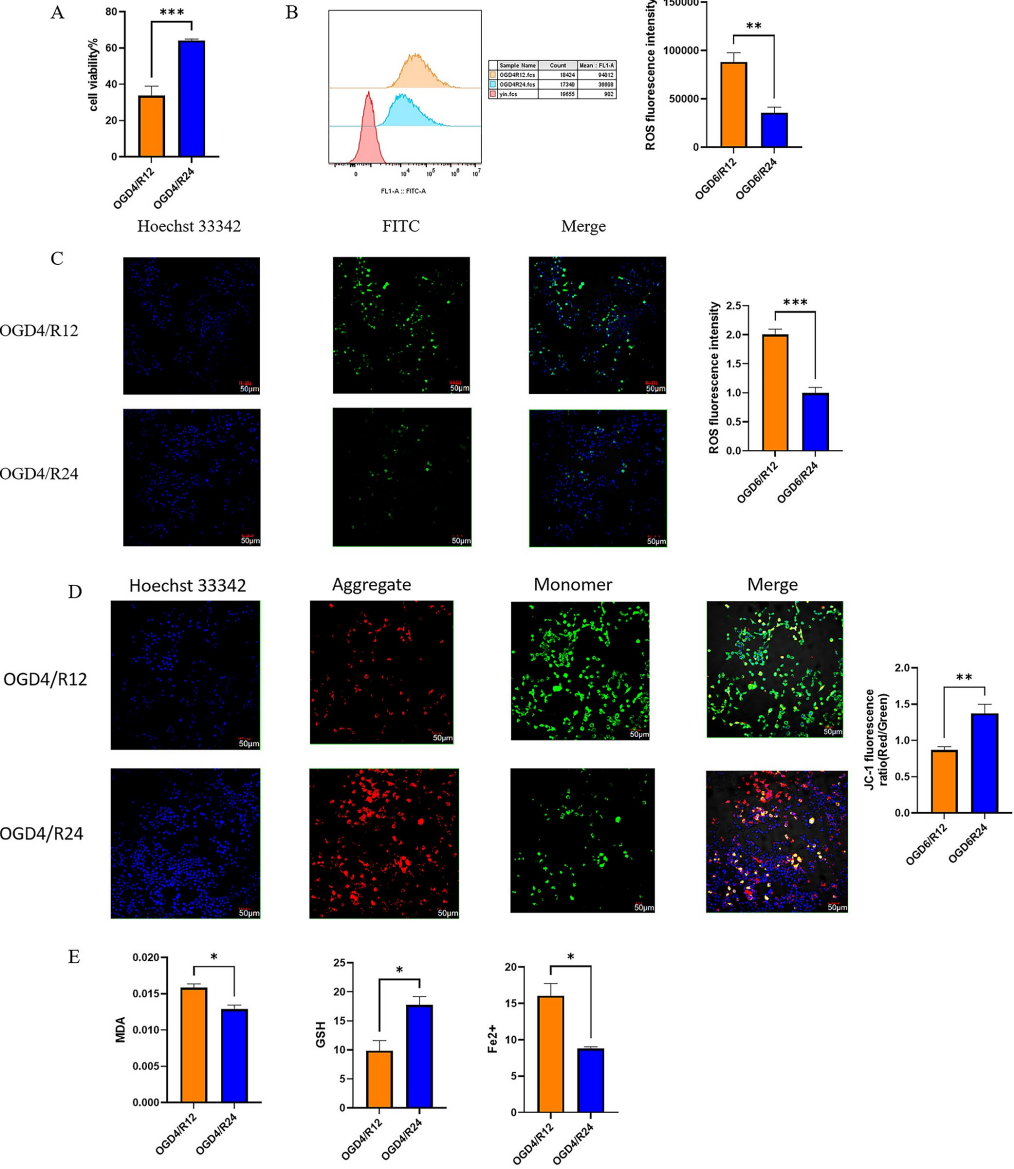
SY5Y were hypoxic for 4 hours and reoxygenated for 12 or 24 hours to establish the OGD/R cell model. The OGD4/R12 group of SY5Y cells was more severely damaged and had more pronounced ferroptosis than the OGD4/R24 group. A. Use the CCK8 Kit to determine cell viability. Cell viability of OGD4/R12 group and OGD4/R24 group. B. Intracellular ROS were detected using DCFH-DA fluorescence assay. ROS detected with flow cytometry. C. Confocal microscopy images and fluorescence intensity of intracellular ROS detected by DCFH-DA fluorescence assay. The scale bar represents 50 μM. D. Confocal microscopy images and JC-1 fluorescence ratio of MMPs detected by the JC-1 kit. Increased red fluorescence indicated the formation of JC-1 aggregates, suggesting a relatively intact mitochondrial membrane. Enhanced green fluorescence indicated the production of JC-1 monomer, suggesting mitochondrial membrane disruption. The scale bar represents 50 μM. E. Detection of MDA, GSH and Fe2+ levels in OGD4/R12 group and OGD4/R24 group by kit. (*p<0.05, **p<0.01, ***p<0.001 OGD4/R24 compared with OGD4/R12, In vitro experiments repeated three times).

CCK-8 results showed that cell viability of SY5Y cells in the OGD4/R12 group was significantly decreased compared to the OGD4/R24 group (Fig 10A). Lipid peroxidation and iron ion accumulation are two important features of ferroptosis. GSH is the main antioxidant and is oxidised from reduced GSH to GSSG in ferroptosis. Mitochondrial membrane potential decreases in ferroptosis. We examined ROS by flow cytometry and confocal microscopy, respectively, and showed an increase in ROS in the OGD4/R12 group compared to the OGD4/R24 group (Fig 10B and 10C). The occurrence of ferroptosis is closely linked to changes in mitochondrial membrane potential. In normal cells, the mitochondrial membrane potential is high and JC-1 is present in the mitochondrial matrix in the form of multimers, producing red fluorescence; in ferroptosis, the mitochondrial membrane potential decreases and JC-1 is present in the mitochondrial matrix in the form of monomers, producing green fluorescence. JC-1 results showed that mitochondrial membrane potential was reduced in the OGD4/R12 group compared to the OGD4R24 group (Fig 10D). In comparison with the OGD4/R24 group, the OGD4/R12 group had higher levels of MDA and Fe^2+^ and lower levels of GSH (Fig 10E).

### 4.9 Expression validation of MAPK3 and ferroptosis genes in SY5Y OGD/R cell model

**Fig 11:**
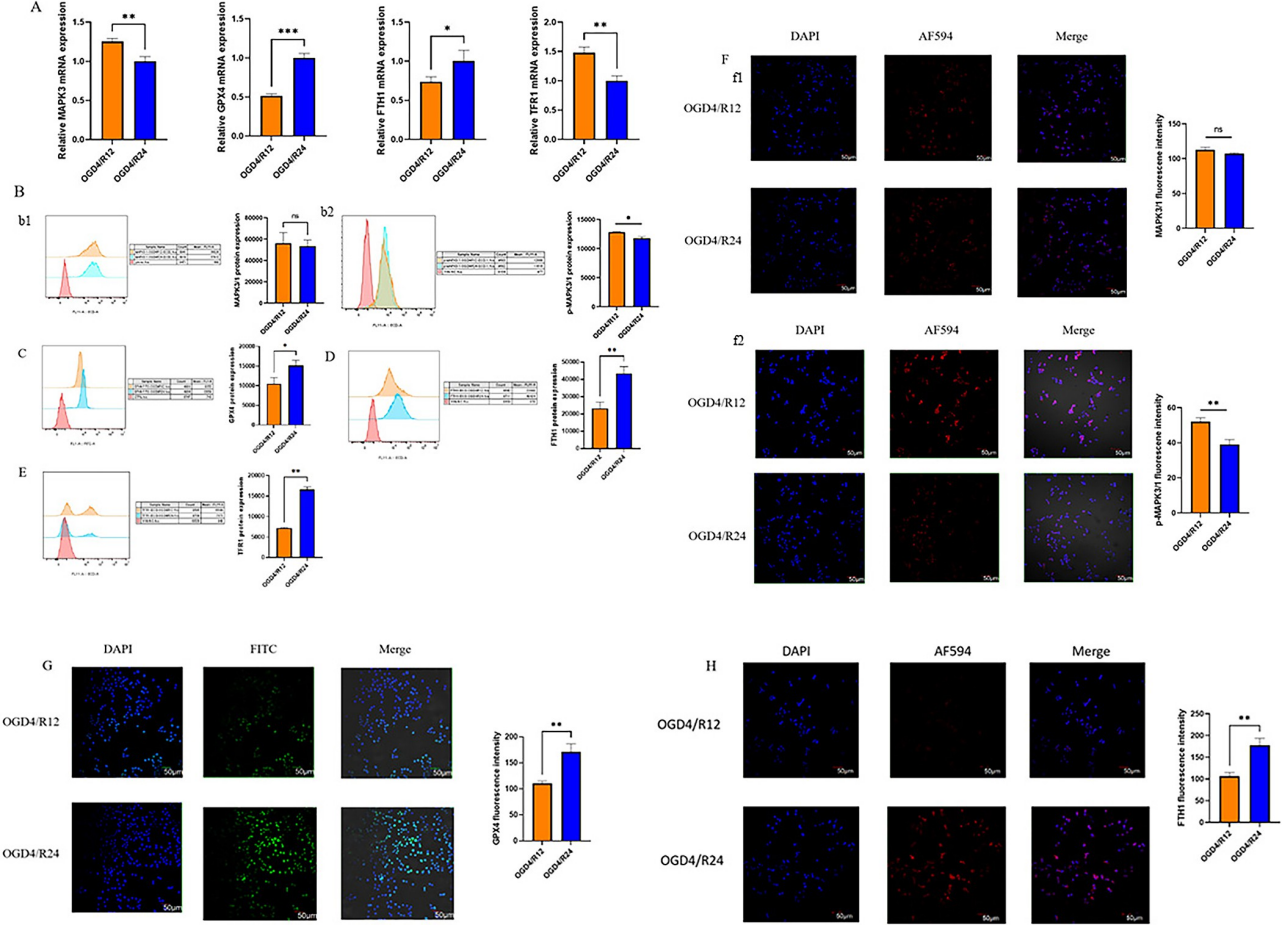
SY5Y were hypoxic for 4 hours and reoxygenated for 12 or 24 hours to establish the OGD/R cell model. Expression of the MAPK3 and ferroptosis signature genes at mRNA and protein levels in the OGD4/R12 and OGD4/R24 groups. A. The mRNA level of MAPK3、GPX4、FTH1、TFR1 were measured in the OGD4/R12 and OGD4/R24 groups by qPCR. B-E. The treated cells were incubated with primary or secondary antibodies. The expression of MAPK3/1 (b1), p-MAPK3/1 (b2), FTH1 (D), TFR1 (E) (ECD channel) and GPX4 (D) (FITC channel) protein were detected by flow cytometry. F-H. The treated cells were fixed, permeabilized, blocked and incubated with primary antibodies, secondary antibodies and probes. The expression of the MAPK3 (f1), p-MAPK3/1 (f2), FTH1 (H) (AF594) and GPX4 (G) (FITC) proteins was observed by immunofluorescence. (*p<0.05, **p<0.01, ***p<0.001 OGD4/R24 compared with OGD4/R12, In vitro experiments repeated three times).

To verify the bioinformatics results, we established an OGD/R cell model to simulate cerebral ischemia-reperfusion injury. qPCR and flow cytometry were performed to detect mRNA and protein, respectively. In response to ferroptosis, GPX4 and FTH1 are downregulated and TFR1 is upregulated [43, 44]. As shown in Fig 11A, compared to OGD4/R12 group, the mRNA expression levels of MAPK3, TFR1 were downregulated in OGD4/R24, the mRNA expression levels of GPX4, FTH1 were upregulated in OGD4/R24. Flow analysis results showed that p-MAPK3/1 and TFR1 protein expression was increased and GPX4 and FTH1 protein expression was reduced in the OGD4/R12 group compared to the OGD4/R24 group. There was no significant change in MAPK3/1 protein expression (Fig 11B–11E). Immunofluorescence experimental results were consistent with flow cytometry (Fig 11F–11H).

## 5. Discussion

Despite significant progresses in cardiac arrest resuscitation and post-cardiac arrest care acquired, mortality maintains high. The primary cause of death in cardiac arrest survivors is severe hypoxic ischemic brain injury [45]. Consequently, neurological prediction has become particularly important in post-cardiac arrest care. More and more research on alleviating hypoxic ischemic brain, the most common study is temperature control [46]. Accumulating evidence demonstrates that prognostic biomarkers after cardiac arrest have been widely researched in recent years, including neuron specific enolase (NSE), S-100B, neurofilament light (NfL), ubiquitin carboxy-terminal hydrolase L1 (UHC-L1), glial fibrillary acidic protein (GFAP) and tau protein [47]. Recent research has demonstrated that miR-9-3p is intensively related to neurological outcome after Cardiac Arrest [48]. European Resuscitation Council and European Society of Intensive Care Medicine guidelines recommend a multimodal neuroprognostic strategy [49]. Identifying a novel biomarker that can preferably evaluate prognosis remains a challenge for clinicians.

Ferroptosis, which has been studied extensively in recent years, is a unique form of iron-dependent programmed cell death. There is increasing evidence indicates that ferroptosis is significantly related to brain injury after cardiac arrest. A recent study demonstrated that cerebral tissue underwent cell ferroptosis after CA resuscitation, mesenchymal stem cells (MSCs) could ameliorate cerebral outcomes after CA resuscitation via reducing cell ferroptosis [50]. Zhou Ye et al. suggested that baicalein mitigates brain injury in Sprague-Dawley (SD) rats after ROSC by inhibiting the ferroptosis and endoplasmic reticulum (ER) stress [51]. The mechanism by which ferroptosis affects brain injury after CA remains unclear.

This study aims to screen the ferroptosis-related genes associated with brain injury after cardiac arrest using bioinformatics technology to provide additional insight into the assessment of neurological outcome. 112 ferroptosis-related genes were selected via intersecting DEGs from GSE29540 and the FerrDb dataset. KEGG enrichment analysis demonstrated that these ferroptosis-related DEGs were significantly enriched in Ferroptosis, Apoptosis, IL-17 signalling pathway, Autophagy. Subsequently, the top 10 hub genes were selected via the MCC algorithm of the Cytoscape CytoHubba plug-in, including HIF1A, MAPK3, PPARA, IL1B, PTGS2, RELA, TLR4, KEAP1, SREBF1, SIRT6. Validated by GSE92696, only the MAPK3 gene remained. ROC analysis demonstrated that the AUC values of the MAPK3 in GSE29540 and GSE92696 were 0.654 and 0.850, respectively.

Mitogen-activated protein kinases (MAPKs), which can adjust proliferation, differentiation and stress response of cellular processes, are a group of signalling proteins [52]. MAPKs mainly consist of four subfamilies: (I) extracellular signal-regulated kinase 1/2 (ERK1/2, MAPK1/3), (II) c-Jun-N-terminal kinase 1–3 (JNK1-3, MAPK8/9/10), (III) p38MAPKα-δ (p38α-δ, MAPK14/11/12/13), and (IV) ERK5 (BMK1, MAPK7) cascades [53]. In datasets GSE29540 and GSE92696, neither MAPK1 nor MAPK4-14 expression was different except MAPK3 expression (S2 and S3 Figs in **S1 File**). One of the MAPKs, MAPK3 is also known as extracellular signal-regulated kinase 1 (ERK1). cellular stimuli like cytokines and the ERK1/2 epidermal growth factor can simultaneously activate ERK1/2. The activation of ERK1/2 causes neurological abnormalities. ERK-1/2 is mainly expressed in cells and is abundantly activated in central nervous system neurons. ERK1/2 is activated during ischemic injury and aggravates brain injury [54]. Extracellular signal-regulated kinases 1 and 2 (ERK1/2) have been implicated in ischemia-reperfusion injury [55, 56]. ERK1/2 can be detected throughout the body and are involved in the Ras-Raf-MEK-ERK signal transduction cascade. The Ras-Raf-MEK-ERK signaling cascade changes in a multiple of diseases, including brain injury [57]. There is increasing evidence that ERK1/2 is closely related to cerebral ischemia-reperfusion injury [58–62]. Several bioinformatics studies have shown that MAPK3 expression is an important biomarker for diagnosis and prediction of disease models [63–65]. Research has shown that upregulation of MAPK3 decreases ischemia/reperfusion induced myocardial apoptosis [66]. Previous research has confirmed that microRNA-15b suppresses Bcl-2 and MAPK3 to aggravate cardiomyocyte apoptosis caused by hypoxia/reoxygenation [67]. In this study, cell viability was decreased in the OGD4/R12 group compared to the OGD4/R24 group, corresponding to CPC 1–2 and CPC 3–5, respectively. Consistent with previous studies the results of the present study showed that MAPK3 phosphorylation may influence ferroptosis [58, 59]. Results from cellular experiments showed that MAPK3 expression was positively correlated with ferroptosis in the OGD4/R12 group in comparison to the OGD4/R24 group. In conclusion, downregulation of MAPK3 expression may suppress neuronal ferroptosis and thus ameliorate neurological outcome after CA. In the near future we will conduct animal studies to verify these findings. The result of miRNAs associated with hub genes indicates that hsa-miR-214-3p and hsa-miR-483-5p are able to regulate the expression of MAPK3. The above result was conducive to exploring the detailed molecular mechanism.

Recent studies has reported that neuronal injury is related to systemic immune cell invasion after CA resuscitation [68]. According to the report,immune dysfunction occurs after CA [69]. Study suggests CD8 T cells may be linked to neurological dysfunction in people with Alzheimer’s disease [70]. Immune infiltration analysis indicated that CD8 T cells, resting CD4 memory T cells, activated CD4 memory T cells, activated NK cells, M1 macrophages in the low CPC group were significantly different from those in the high CPC group. In correlation analysis, we demonstrated that MAPK3 was positively correlated with naive B cells, M0 macrophages, activated dendritic cells and negatively correlated with activated CD4 memory T cells, CD8 T cells, memory B cells. To sum up, the immune response affects the neurological prognosis of cardiac arrest, but the molecular mechanism remains unclear.

Nevertheless, our study has some shortcomings. Firstly, datasets on cardiac arrest are limited, furthermore, different sources of datasets may influence the final results. On the other hand, it is also important to note that our study lacked in vivo experiments, and in the future we will need animal experiments to simulate the construction of the CA/CPR model to further investigate the above findings. we need to carry out more basic experiments for further research in the future.

## 6. Conclusion

In summary, the MAPK3 ferroptosis-related gene could be used as a biomarker to predict the neurological outcome after cardiac arrest. Potential biological pathways provide new insights into the pathogenesis of cardiac arrest.

## Supporting information

S1 File (DOCX)

S1 Data Genes associated with ferroptosis. (XLS)

S2 Data diffGeneExp. (XLS)

S3 Data diff. (XLS)

S4 Data Ferroptosis-related differential genes. (XLS)

S5 Data miRDB. (XLS)

S6 Data miRTarget. (XLS)

S7 Data starBase v2.0. (XLS)

S8 Data vennData. (XLS)
