# Supplementary material for: Ferroptosis-related gene MAPK3 is associated with the neurological outcome after cardiac arrest
Source: PLoS One. 2024 Jun 17;19(6):e0301647. doi: 10.1371/journal.pone.0301647 (PMC11182507; doi:10.1371/journal.pone.0301647)

Supplementary fig1: (A-J) Expression of hub genes in GSE29540


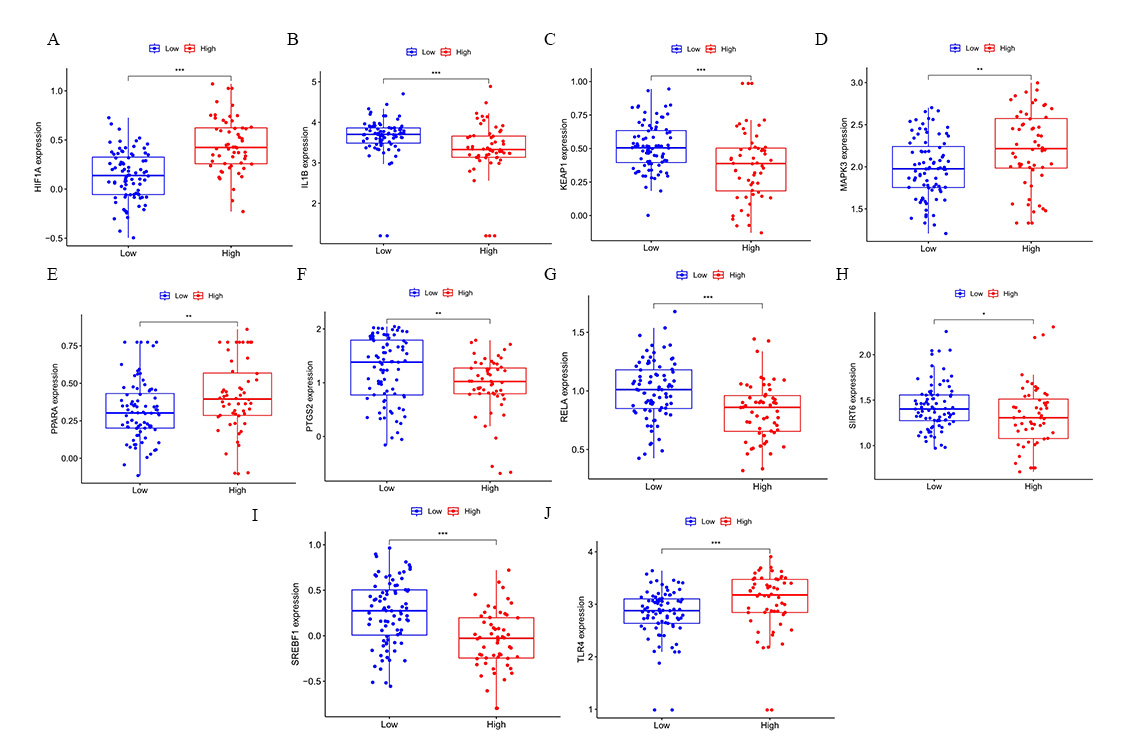


Supplementary fig 2：(A-K) MAPK1, MAPK4-14 expression in GSE29540

GSE29540


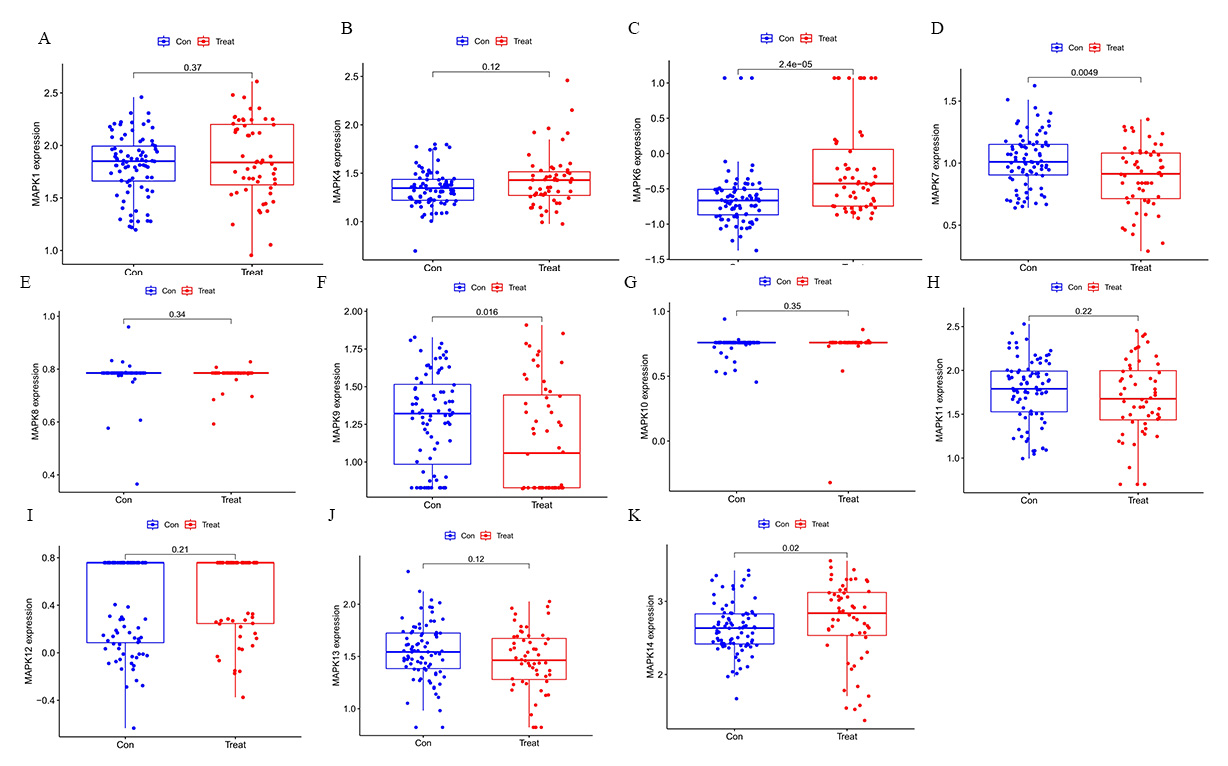


Supplementary fig 3：(A-K) MAPK1, MAPK4-14 expression in GSE92696


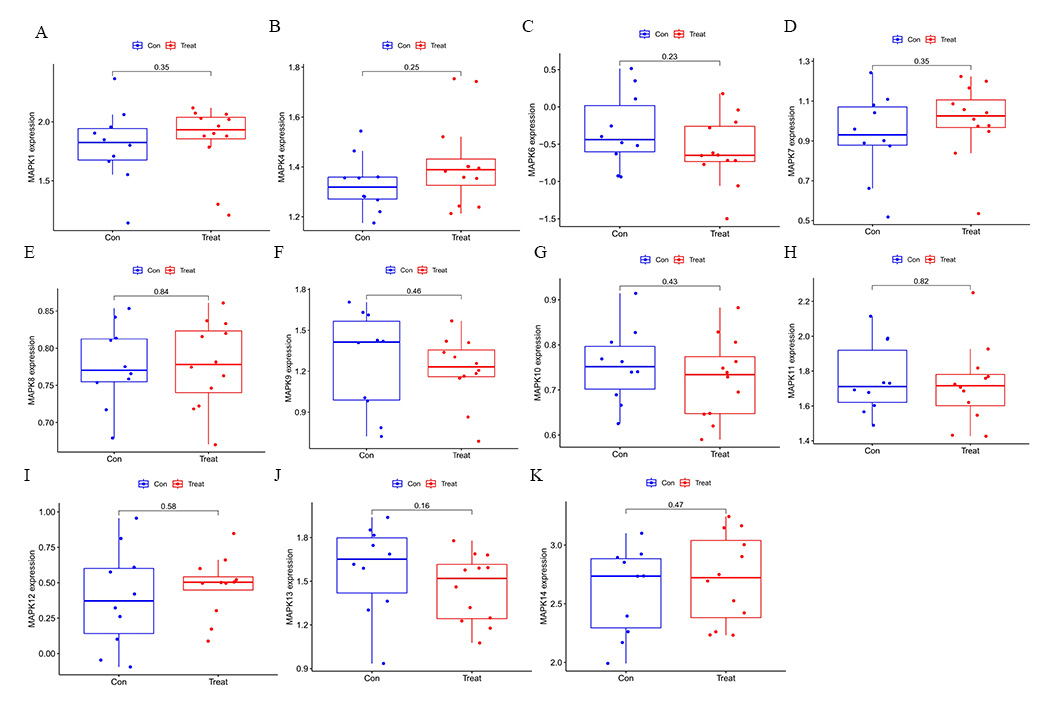

Supplement: S1 File — (DOCX) [file pone.0301647.s001.docx]
